# Complex population genetic and demographic history of the Salangid, *Neosalanx taihuensis*, based on cytochrome b sequences

**DOI:** 10.1186/1471-2148-8-201

**Published:** 2008-07-14

**Authors:** Liang Zhao, Jie Zhang, Zhijin Liu, Stephan M Funk, Fuwen Wei, Muqi Xu, Ming Li

**Affiliations:** 1 Key laboratory of Animal Ecology and Conservation Biology, Institute of Zoology, Chinese Academy of Sciences, Chaoyang, Beijing 100101, PR China; 2 Key laboratory of zoological Evolution and Systematics, Institute of Zoology, Chinese Academy of Sciences, Chaoyang, Beijing 100101, PR China; 3 Faculty of biology, Suzhou University, Suzhou, Anhui 234000, PR China; 4 Graduate School of the Chinese Academy of Sciences, Beijing 100039, PR China; 5 Nature Heritage Ltd, London, UK

## Abstract

**Background:**

The Salangid icefish *Neosalanx taihuensis *(Salangidae) is an economically important fish, which is endemic to China, restricted to large freshwater systems (e.g. lakes, large rivers and estuaries) and typically exhibit low vagility. The continuous distribution ranges from the temperate region of the Huai and Yellow River basins to the subtropical region of the Pearl River basin. This wide ranging distribution makes the species an ideal model for the study of palaeoclimatic effects on population genetic structure and phylogeography. Here, we aim to analyze population genetic differentiation within and between river basins and demographic history in order to understand how this species responded to severe climatic oscillations, decline of the sea levels during the Pleistocene ice ages and tectonic activity.

**Results:**

We obtained the complete mtDNA cytochrome *b *sequences (1141 bp) of 354 individuals from 13 populations in the Pearl River, the Yangze River and the Huai River basin. Thirty-six haplotypes were detected. Haplotype frequency distributions were strongly skewed, with most haplotypes (n = 24) represented only in single samples each and thus restricted to a single population. The most common haplotype (H36) was found in 49.15% of all individuals. Analysis of molecular variance (AMOVA) revealed a random pattern in the distribution of genetic diversity, which is inconsistent with contemporary hydrological structure. Significant levels of genetic subdivision were detected among populations within basins rather than between the three basins. Demographic analysis revealed that the population size in the Pearl River basin has remained relatively constant whereas the populations in the Yangze River and the Huai River basins expanded about 221 and 190 kyr ago, respectively, with the majority of mutations occurring after the last glacial maximum (LGM).

**Conclusion:**

The observed complex genetic pattern of *N. taihuensis *is coherent with a scenario of multiple unrelated founding events by long-distance colonization and dispersal combined with contiguous population expansion and locally restricted gene flow. We also found that this species was likely severely impacted by past glaciations. More favourable climate and the formation of large suitable habitations together facilitated population expansion after the late Quaternary (especially the LGM). We proposed that all populations should be managed and conserved separately, especially for habitat protection.

## Background

Population genetic structure is dependent on the interaction of the biology of a species and the environment in which it resides. Marine organisms generally show low levels of genetic differentiation over large geographic distances [1,2]. In open marine systems it is evident that higher dispersal potential during planktonic egg, larval, or adult stages coupled with the absence of physical barriers to movement seems to greatly facilitate extensive gene flow among populations of organisms [3,4]. However, populations of freshwater fish species from different basins often show significant genetic differentiation resulting from isolation, while populations within a basin often show no or low levels of genetic differentiation [5-9]. There is evidence that for some species instream barriers can restrict gene flow and induce high levels of genetic differentiation between populations [10,11]. Even in the absence of restricted gene flow, multiple unrelated founding events could also shape the same pattern of population genetic structure [4].

Due to periodic climatic oscillations during the Pleistocene, range contractions, range expansions and changes in interconnectivity within and between drainages during the periodic climatic oscillations of the Pleistocene have greatly influenced the distribution of many fish species, have significantly altered the amount and distribution of intraspecific genetic variation in many species and have remodelled the population structures of temperate fish faunas [1,5]. The major climatic oscillations occurred during the past 800 kyr (with a 100 kyr dominant cycle), and the last glacial maximum (LGM) occurred about 18 kyr ago with a decline of the sea levels of about 120–140 m [12]. Each of the severe climatic shifts and the change in the sea levels could have produced great changes in the freshwater fish species' geographical distribution and abundance [1,5,13,14]. The legacy of these changes in the phylogeographic distribution of genetic diversity has been traced in several species [5,15,16]. In Cyprinids, it has been shown that glacial, interglacial and postglacial oscillations have resulted in major changes of distributions and have shaped current distributions by changes of river courses and river confluences in response to falling sea levels [13,14].

The Family Salangidae, which is classified as Protacanthopterygii, Order Osmeriformes, Suborder Osmeroidei [17,18], comprises six genera and approximately 17 species [19,20]. The Salangid, *Neosalanx taihuensis*, is one of the most economically important species in this Family. This species is endemic to China and is restricted to large bodied freshwater systems such as inland lakes, out-flowing rivers and estuaries, covering a continuous distribution from the temperate (Yellow River basin) to the subtropical zone (Pearl River basin) [21]. Also, this species exhibits annualism poor swimming ability and has some neotenic features such as transparent miniaturized bodies, cartilaginous endoskeletons, and notochords [20,22]. The combination of relative large spatial distribution encompassing discrete drainages in tropical and subtropical regions and the low vagility constitutes a good model system to study the palaeoclimatic effects on population genetic structure and phylogeography of freshwater [23]. In recent years, because of water pollution, overfishing and habitat destruction, the population of *N. taihuensis *has undergone the lost of appropriate habitat and the decrease in population size [24,25]. In order to protect the resource of *N. taihuensis*, the habitat detection has to be implemented immediately, and the study on the *N. taihuensis *population structure and its phylogeoraphic pattern should be enhanced since the failure to detect population units will lead to local overfishing and ultimately to severe declines [26]. The Salangidae family has been studied for more than 200 years, but most of studies have focused on taxonomy [27,28], biology [29], and molecular phylogeny [30]. Previous studies in China have shown that in two unrelated species,*Opsariichthys bidens *(Teleostei, Cyprinidae) and *Brachymystax lenok *Pallas (Salmoninae, Salmonidae), both had genetic patterns coinciding with contemporary drainage structure [8,9], but the related study on *N. taihuensis *is not conducted till now.

**Figure 1:**
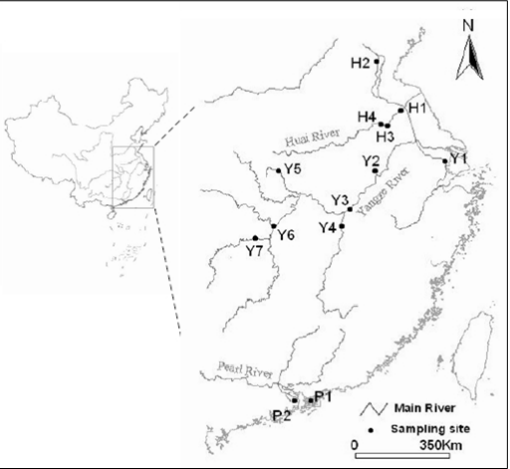
Map of the distribution of *N. taihuensis *and the sampling locations for this study.

The basins of Yangtze River, Huai River and Pearl River, in which *N. taihuensis *mainly distributes, are important river systems in China. Although a strong uplifting of the Tibetan plateau occurred during the Pleistocene in southwest China [31], there is no evidence to indicate that large-scale river rearrangement occurred after the Pliocene in the mid-lower reaches of these basins [32]. The Yangze River and the Huai River basin are connected by the Peking Hangzhou Canal, while there are no connection between Pearl River basin and the others[33]. Given the intolerance of the *N. taihuensis *to waters with some salt content, members of freshwater fish division cannot disperse by river connections [34]. Therefore, we expect vicariance among basins might play an important role in shaping population genetic structure of *N. taihuensis*. In addition, the glaciations during the Pleistocene, especially the last glacial maximum (LGM) with sea levels lowered (120–140 m below present sea level), should have severely affected the genetic structure of this species. In order to detect the population structure and phylogeographic pattern of *N. taihuensis *at large geographical scales, samples of *N. taihuensis *were collected from 13 populations among the Pearl River basin, the Yangtze River basin, and the Huai River basin (Figure. 1). The complete mtDNA cytochrome *b *gene (*cyt b*) sequence was used to analyze population genetic differentiation of this species, population dynamics and demographic history, and to show how the glaciations influenced the phylogeographic patterns of freshwater fish, and how this species responded to the severe climatic oscillations and decline of the sea levels during the Pleistocene ice ages.

## Results

### Sequence Variation and Genetic Diversity

For 1141 bp of the complete *cyt b *gene analyzed in 354 individuals, there were 38 variable nucleotide sites, and no insertions or deletions in any of the sequences. The average nucleotide composition for all individuals was highest for C (0.334), followed by T (0.277), A (0.214), and G (0.175). Nucleotide composition showed an anti-G bias (G = 17.5%), which is characteristic for the mitochondrial genome [35]. Since there were no stop codons when the *cytb *sequence was translated into amino acid sequences, and based on the criteria of Zhang & Hewitt [36], we found no evidence for nuclear mitochondrial pseudogenes (Numts) in our PCR sequences. Linkage disequilibrium (LD) test and selective neutrality test were implemented for *cyt b *sequences in *N. taihuensis*, which indicated that there was no signs of recombination (*P *= 0.925) and the neutral hypothesis (no selection) could not be rejected (*P *= 0.172) for these *cyt b *sequences. Thus, the *cyt b *was suitable marker for analyzing the evolutionary history of this species.

Thirty-six different haplotypes were identified in the 354 samples analyzed (Table 1 and Table 2). The number of haplotypes ranged from 5 to 25 for each basin. The populations with the greatest number of haplotypes were the Taihu lake population (8) and Chaohu lake population (8) in Yangze River basin, and the Hongzehu lake population (8) in Huai River basin. The haplotype frequency distribution was strongly skewed, with the vast majority of haplotypes found only once (24 out of 36) and restricted to a single population. H36, the most common haplotype found in 12 sampled populations with the exception of the Xujiahe reservoir population, occupied 49.15% of all individuals among all populations (56.82% for the Pearl River basin, 37.43% for the Yangtze River basin, 72.57% for the Huai River basin). Other common haplotypes found in all three basins were H27, H33 and H25, with 16.38%, 10.73% and 3.11% of all individuals, respectively (Table 2).

**Table 1:** Descriptive statistics of *N. taihuensis *phylogroups based on mitochondrial *cytb *sequence data

Population	location	*N*	*H*	*h*	*S*	*k*	*π*
**Pearl River basin**		44	5	0.622 ± 0.064	8	1.261	0.0011 ± 0.0003
P1	Luofu River	31	5	0.443 ± 0.105	8	1.213	0.0011 ± 0.0004
P2	Pearl River	13	3	0.513 ± 0.144	2	0.744	0.0007 ± 0.0002
**Yangze River basin**		197	25	0.781 ± 0.017	28	3.080	0.0027 ± 0.0001
Y1	Taihu lake	31	8	0.798 ± 0.039	11	3.174	0.0028 ± 0.0002
Y2	Chaohu lake	25	8	0.763 ± 0.068	10	1.380	0.0012 ± 0.0003
Y3	Bohu lake	29	5	0.650 ± 0.078	10	2.606	0.0023 ± 0.0004
Y4	Poyanghu lake	22	5	0.407 ± 0.128	8	1.589	0.0014 ± 0.0005
Y5	Dongtinghu lake	27	5	0.393 ± 0.114	6	0.576	0.0005 ± 0.0002
Y6	Tianrezhou	34	5	0.570 ± 0.078	8	1.210	0.0011 ± 0.0003
Y7	Xujiahe Reservoir	29	2	0.296 ± 0.093	3	0.887	0.0008 ± 0.0002
**Huai River basin**		113	15	0.464 ± 0.057	18	1.110	0.0010 ± 0.0002
H1	Hongzehu lake	26	8	0.686 ± 0.088	11	2.348	0.0021 ± 0.0003
H2	Weishanhu lake	31	4	0.553 ± 0.060	6	0.796	0.0007 ± 0.0002
H3	Wabuhu lake	30	2	0.067 ± 0.061	1	0.067	0.0001 ± 0.0001
H4	Chengdonghu lake	26	6	0.354 ± 0.119	9	0.834	0.0007 ± 0.0003
**Entire region (all samples)**		354	36	0.713 ± 0.022	38	2.528	0.0022 ± 0.0001

Note:*N*, number of individuals; *H*, number of haplotypes; *S*, number of segregating sites; *h*, gene diversity (± Standard Deviation); *k*, mean pair-wise nucleotide; *π*, nucleotide diversity (± Standard Deviation)

**Table 2:** Summary of mtDNA *cytb *region haplotype distributions

Haplotypes\populations	Pearl River basin		Yangze River basin							Huai River basin				Total
		
	P1	P2	Y1	Y2	Y3	Y4	Y5	Y6	Y7	H1	H2	H3	H4	
**H01**	1													1
**H02**	3													3
**H03**			1											1
**H04**			1											1
**H05**			1											1
**H06**			1											1
**H07**			2											2
**H08**				1										1
**H09**				1										1
**H10**				4										4
**H11**				1										1
**H12**					2									2
**H13**					1									1
**H14**						1								1
**H15**						1								1
**H16**							1							1
**H17**							3							3
**H18**							1							1
**H19**							1							1
**H20**								1						1
**H21**								1						1
**H22**										1				1
**H23**				1						1				2
**H24**										1				1
**H25**										2				2
**H26**										1				1
**H27**			9		16			3	24	5	1			58
**H28**											1			1
**H29**	1	9				2		8			11			31
**H30**												1		1
**H31**													1	1
**H32**													1	1
**H33**			7	1	6	17			5	1			1	38
**H34**													1	1
**H35**	3	2		5									1	11
**H36**	23	2	9	11	4	1	21	21		14	18	29	21	174
**Sample Number**	31	13	31	25	29	22	27	34	29	26	31	30	26	354

Diversity indices (average ± standard deviation), *h *and *π *, are summarized in Table 1. The total of *h *was 0.713 ± 0.022 ranging from 0.473 ± 0.057 to 0.781 ± 0.017, and *π *was 0.0022 ± 0.0001 ranging from 0.0010 ± 0.0002 to 0.0027 ± 0.0001 among there basins. There are no evidences that heterogeneous levels of genetic variability were produced by unequal sample size for each population through Pearson's correlation test between diversity indices and sample size (r = 0.082 and *P *= 0.790 for numbers of haplotypes, r = -0.02 and *P *= 0.996 for haplotype diversity, and r = 0.141 and *P *= 0.645 for nucleotide diversity, respectively). These results showed a medium/high haplotype diversity and a low nucleotide diversity, and that the genetic diversity for three near-coastal populations (the Taihu lake population and the Chaohu lake population within the Yangze River basin, and the Hongzehu lake population within the Huai River basin) were higher than others (Table 1).

### Population Structure and Phylogeography

**Figure 2:**
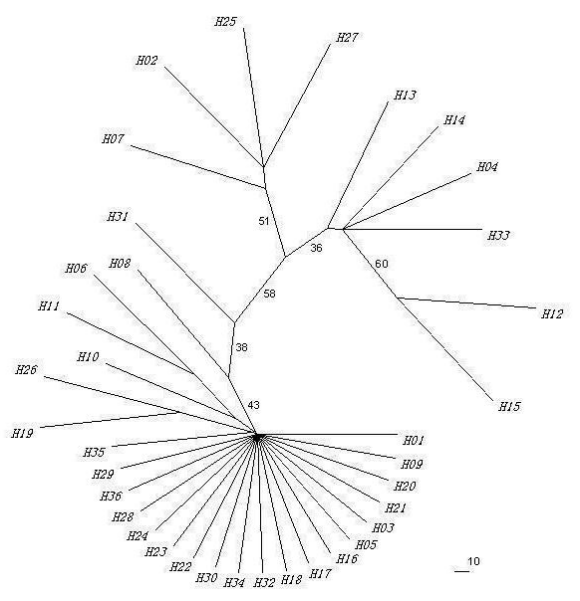
**Unrooted Maximum-likelihood tree for all 36 haplotypes of *N. taihuensis***. Labels are haplotype identification numbers shown in Table 2. Values indicate bootstrap support for each node based on maximum likelihood inference.

**Figure 3:**
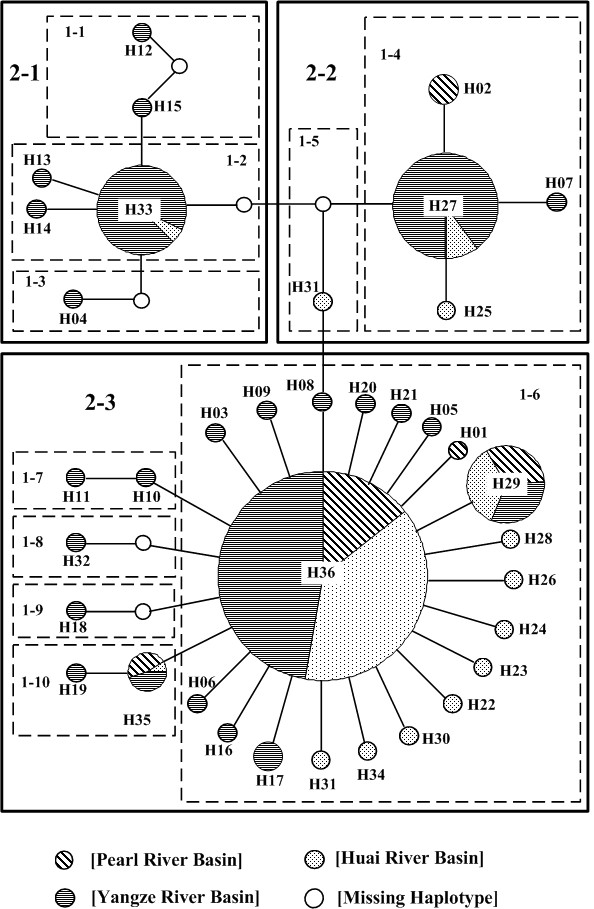
**Minimum spanning network based on Templeton *et al*. (1987, 1993) statistical parsimony**. Nodes contain the haplotype number and are proportional to the haplotype frequency. White nodes indicate undetected intermediate haplotype states separated by one mutational step. Boxes indicate one-step to two-step nesting levels for the nested clade analysis in NCA.

Unrooted ML tree revealed that there were no distinct haplotype groups with high bootstrap support (<60 %) and there was no geographical structure among haplotypes (Figure. 2), which was supported by network analysis (Figure. 3). In this study, only a few missing haplotypes were detected, which indicated that we gathered enough samples (both numbers of individuals and locations within the distribution) for phylogeographical analysis. Each haplotype was connected with others by 1 to 3 mutational steps, suggesting that there was no deep branching among halotypes for this species. In network, H36 yielded the highest outgroup weight (0.2941) followed by H27 (0.0588), H33 (0.0588), and H35 (0.0441), indicating H36 was the most probable ancestral haplotype [37]. In fact, the high frequency of shared haplotypes (i.e., H36, H27, H33, H29) were found among all basins, which produced a pattern of population relationships associated with the ancestry of the haplotypes found within each population rather than with the hydrological network (Figure. 2, Figure. 3). Thus, we suggested that there was no obvious phylogeographic pattern within *N. taihuensis*.

Pair-wise comparisons *Φ*_*ST *_test among 13 populations showed that the differences among the most of populations were irrelevant to their distributions (Table 3). AMOVA analysis revealed that there were significant subdivisions within and among populations of *N. taihuensis*, but no significant genetic variance was found among basins, with only 8.43% of the genetic variance was found among basins (Table 4), which provided evidences that the high level of population structure was not related to the hydrological pattern. The Mantel test indicated no significant relationship between Φst/(1-Φst) and geographic distance among populations, with *P *= 0.59 for all 13 populations among basins, *P *= 0.82 for 7 populations within Yangze River basin and *P *= 0.19 for 4 populations within Huai River basin, respectively.

**Table 3:** Pairwise *Φ*_*ST *_among *N. taihuensis *populations

populations	Pearl River basin		Yangze River basin							Huai River basin			
	
	P_1_	P_2_	Y_1_	Y_2_	Y_3_	Y_4_	Y_5_	Y_6_	Y_7_	H_1_	H_2_	H_3_	H_4_
P_1_		61.9	1335.0	1155.4	953.7	808.1	808.5	869.7	1081.9	1429.4	1532.0	1221.0	1193.8
P_2_	0.3146*		1360.6	1176.6	974.9	829.3	777.5	838.7	1074.7	1450.6	1553.2	1215.0	1242.2
Y_1_	0.3081*	0.4161*		331.0	471.7	619.1	983.8	822.0	658.2	278.9	604.6	380.5	423.6
Y_2_	0.0309	0.2873*	0.3292*		194.9	344.0	645.9	484.1	328.8	261.2	462.1	108.8	125.1
Y_3_	0.5349*	0.6046*	0.0511	0.5442*		142.3	513.0	351.1	333.4	470.5	600.8	268.1	259.2
Y_4_	0.7094*	0.7592*	0.2791*	0.6947*	0.2818*		413.4	291.8	389.6	618.0	720.6	409.6	382.4
Y_5_	0.0420	0.4304*	0.4070*	0.0633*	0.6241*	0.7834*		156.4	419.6	885.8	951.7	658.0	626.7
Y_6_	0.0340	0.1611	0.3255*	0.0817*	0.5451*	0.7073*	0.0768*		271.5	723.9	789.8	496.1	464.8
Y_7_	0.7476*	0.8327*	0.2340*	0.7560*	0.0675	0.5831*	0.8353*	0.7463*		560.0	500.8	333.8	276.9
H_1_	0.0753	0.2961	0.0996	0.1331	0.3043*	0.5349*	0.1874*	0.1016	0.5247*		316.1	224.9	291.3
H_2_	0.1196*	0.1137	0.4003*	0.1459*	0.6103*	0.7622*	0.1541*	-0.0008	0.8109*	0.1988*		351.8	338.1
H_3_	0.0582	0.6489*	0.4499*	0.1012*	0.6725*	0.8403*	0.0300	0.0927*	0.8882*	0.2283*	0.2123*		53.6
H_4_	-0.0008	0.3672*	0.3368*	0.0295	0.5673*	0.7339*	0.0169	0.0383	0.7941*	0.1157	0.1212*	0.0190	

*Φ*_*ST *_values are reported below, and the river distances (km) between populations above, the diagonal. Asterisks indicate significant values after Bonferroni correction.

**Table 4:** The results of AMOVA for *N. taihuensis *mtDNA *cytb *estimated using *Φ*-statistics

Source of variation	d.f.	Sum of squares	Variance components	% variation	Φ statistics (P-value)
**Among basins**	2	55.055	0.1143	8.43	Φ_CT _= 0.0843
**Among populations within basins**	10	158.543	0.5593	41.26	Φ_SC _= 0.4506**
**Within populations**	341	232.530	0.6819	50.31	Φ_ST _= 0.4970**
**Total**	353	446.127	1.3555		

(*P < 0.05, ** *P *< 0.01)

The NCA analysis (see additional file 1) provided some historical information for the pattern of genetic differentiation for *N. taihuensis*. The widespread clade 1–6 with significantly small *Dc *and large *Dn*, were indicative long-distance colonization (and/or past fragmentation). Clade 1–2, clade 1–4, and two second cladistic levels of clade 2-1, clade 2-2 indicated restricted gene flow with isolation by distance, and the overall picture of NCA indicated historical contiguous range expansion.

### Demographic Analysis

**Figure 4:**
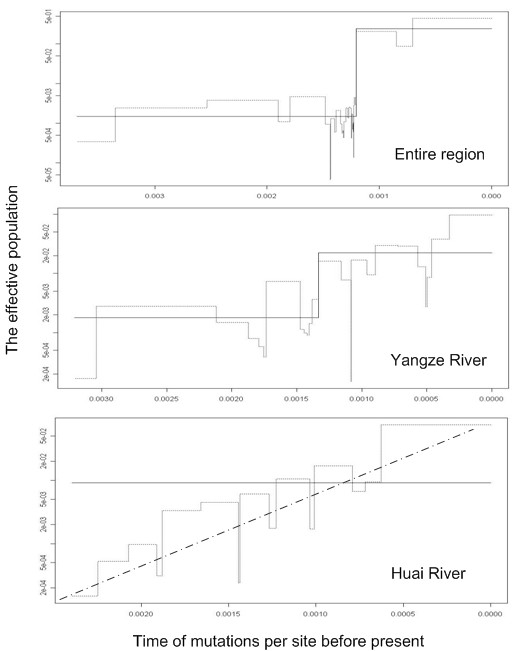
**Skyline plots of changes in effective population size on time**. The *x *axis is time of mutations per site before present; the *y *axis is the effective population size equal to *N*_*e*_*μ*. Classic skyline was shown as thin line, generalized skyline as thick line, and dot line as the expected demographic history.

**Figure 5:**
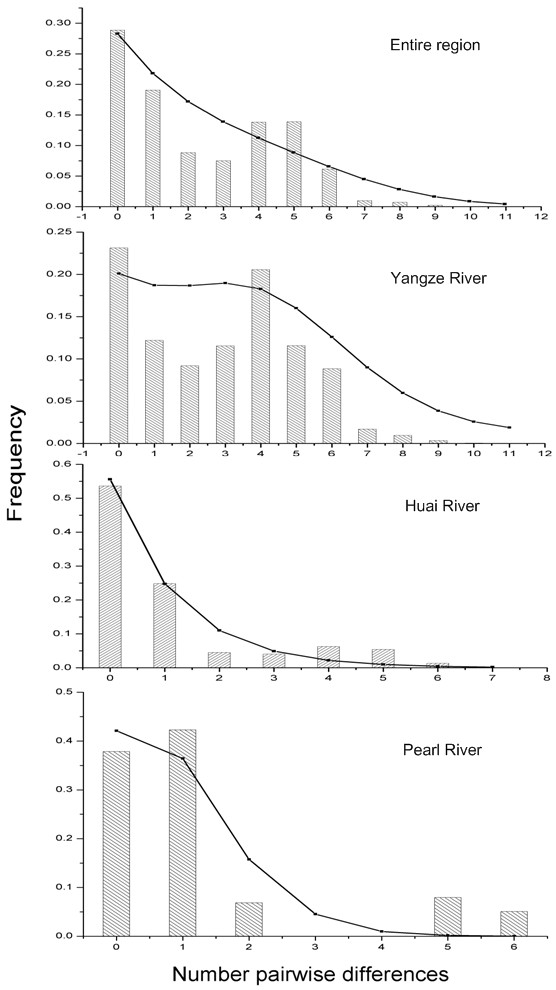
**Observed and expected mismatch distributions showing the frequencies of pairwise differences**. The observed distributions (bars) are compared for their goodness-of-fit to a Poisson distribution under a model of sudden expansion illustrated by the overlaid curve (black dots and solid lines). *X*-axis: number of pair-wise differences, *Y*-axis: frequency.

An examination of demographic histories revealed the marked differences among the basins under study (Table 5, Figure 4, Figure 5). The skyline plots [38,39]of *N. taihuensis *in the entire region and the Yangze River basin showed a sudden stepwise expansion. Fu's *Fs *[40], Ramos-Onsins and Rozas's *R*_2 _[41] tests for the entire region and Fu's *Fs *test for Yangze River basin were statistically significant negative, supported this point. Although both *Fs *and *R*_2 _tests could not effectively tell the causes of bottleneck or expansion, Fu and Li's D* [42] tests were not significant (*P *> 0.05) for these populations, suggesting that there were no historical reduction in effective population size in these regions. The mtDNA mismatch analyses for the entire region and the Yangze River basin showed bimodal profile which might result from constant population size among an old population or an admixture population (Figure 5). However, the mismatch distribution goodness of fit test (Table 5) was not significant, which indicated that there were no severe departure from the estimated demographic model. Moreover, the results of the coalescent based analysis using FLUCTUATE1.4 [43] showed high growth rate, g = 2648.97 ± 556.26 for the entire region and *g *= 3268.21 ± 873.30 for the Yangze River basin, providing powerful evidences of population expansion in these regions. In contrast to that of the entire region and the Yangze River basin, the skyline plot for Huai River basin provided evidence that the exponential growth model fitted the population demographic fluctuation well (Figure 4). Significant Fu's *Fs *(*P *<0.05) and Ramos-Onsins and Rozas's *R*_2 _(*P *< 0.05) test, as well as high growth rate (*g *= 2083.14 ± 529.35) confirmed population growth in Huai River basin, which was also supported by the test of *Hri *(*P *> 0.95), although the bimodal mismatch distribution (Figure. 5) and significance of *SSD *(*P *< 0.05) indicated a poor fit for the stepwise growth model (Table 5). For the Pearl River basin, the bimodal mismatch distribution (Figure. 5) and significance of *SSD *(*P *< 0.05) values indicated a relative constant population size. Fu and Li's D* test, Fu's *Fs *and *R*_2 _test for Pearl River basin were not significant, which also rejected population expansion/bottleneck model (Table 5). In addition, a relative constant population size was confirmed by a relative low growth rate (g = -309.64 ± 955.56) with the approximate 95% confidence interval of *g *included zero in this region.

**Table 5:** Statistical test for neutrality, mismatch analysis and the estimate of demographic parameters for *N. taihuensis *based on mitochondrial *cytb *sequence data

	Entire region	Pearl River basin	Yangze River basin	Huai River basin
*Hri *(***P *value**)	0.0675(0.4687)	0.1418(0.0608)	0.0518(0.2609)	0.1446(0.999)
*SSD *(***P *value**)	0.0388 (0.3002)	0.0239(0.0411) *	0.0336(0.1401)	0.0131(0.001)**
D* (***P *value**)	-0.1047(0.5730)	-0.1036(0.5910)	-0.0991(0.5590)	-0.1276(0.5060)
*R*_2 _(***P *value**)	0.0726(0.0160)*	0.1177(0.1794)	0.0825(0.1430)	0.0859(0.0020)**
*Fs *(***P *value**)	-18.9472 (0.0002)**	0.4106(0.6238)	-7.4204 (0.0279) *	-9.2412 (0.0019) **
*g *± 3*SD	2648.97 ± 556.26	-309.64 ± 955.56	3268.21 ± 873.30	2083.14 ± 529.35
***τ ***(**90%CI**)	5.602 (0.619–10.090)		5.064 (1.334–8.439)	
TMRCA (**kyr**)		73.53		190.0

(*P < 0.05, ** *P *< 0.01)

According to the tau value (τ) and the mutation rates for the *cyt b *gene (1% per nucleotide per generation per million years), the estimated expansion time was found to be 245.5 kyr ago for the entire region and 221.9 kyr ago for the Yangze River basin. Using the coalescent method, an estimate of TMRCA was 190.0 kyr for this species in the Huai River basin and 73.53 kyr in the Pearl River basin, respectively (Table 5).

**Figure 6:**
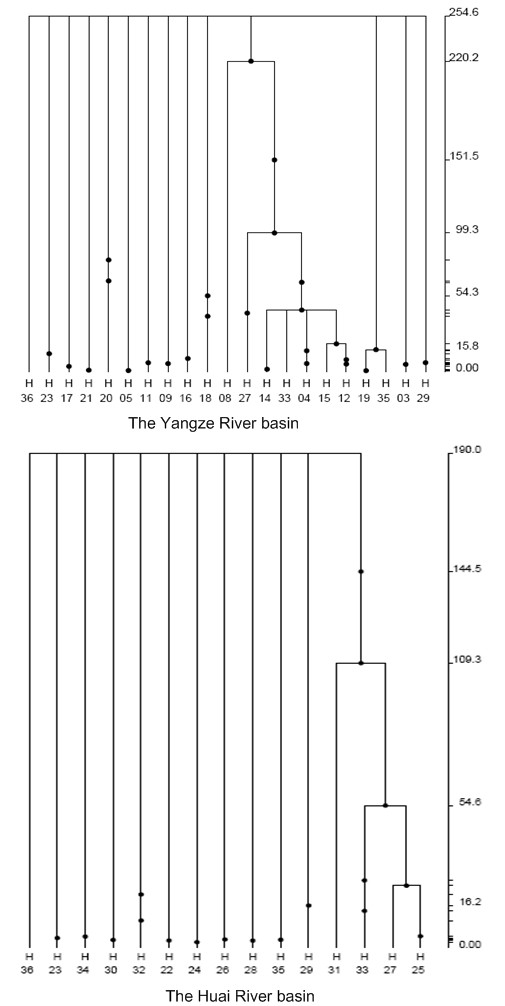
**Gene tree of the *N. taihuensis *mitochondrial *cytb *gene**. The tree is based on 1,000,000 coalescent simulations. The absolute time scale on the right shows the TMRCA (kyr) in generations using a mutation rate of 1% × 10^-6 ^per year. The tree shows the ancestral distribution of mutations and events (TMRCA, recent rapid expansion) in the population history.

To get a more detailed picture of the mutational pattern for population expansion of *N. taihuensis *within the Yangze River basin and the Huai River basin, GENETREE [44] was used to construct a gene tree for determining the distribution of mutation time. To make the sequence data suitable for the infinite-sites model, four haplotypes (H06, H07, H10, and H13) were excluded in this analysis. It is worth to note that the possible removal of data can have important effects on inference, and can lead to unusual conclusions [44], but the results from this procedure can provide a rough profile for the distribution of mutations. As shown in Figure. 6, it was evident that the vast majority of mutations occurred near the tips of the gene tree, with 61.54% mutations occurring within 15.6 kyr in the Yangze River basin and 66.67% mutations occurring within 16.2 kyr in the Huai River basin.

## Discussion

### Population Genetic Structure and Phylogeography

Our results revealed low nucleotide diversity (*π *= 0.0022 ± 0.0001) and medium/high haplotype diversity (*h *= 0.713 ± 0.022) in *N. taihuensis *(Table 1), which could reflect a recent expansion or a short evolutionary history of the population [1,45-47]. This scenario was also supported by the absence of deep branching among haplotypes in the genealogical tree and the "star-like" shaped network (Figure. 2 and Figure. 3) as well as NCA and demographic history analysis (Table 5).

Phylogenetic inference based on unrooted ML tree revealed that there were no distinct clades with high bootstrap support (<60%) and individuals from three basins did not partition into distinct clades (Figure. 2), which indicated no geographical structure among haplotypes. The statistical parsimony network displayed a "star-like" shape with H36 being the most probable ancestral haplotype (outgroup weight: 0.2941) (Figure. 3), which also revealed a lack of geographical structure.

Beyond the prediction that vicariance among basins should play an important role in shaping population genetic structure, the results of AMOVA showed that the contemporary drainage structure served as a poor model in explaining the high levels of genetic differentiation, with only 8.43% variance among basins, it was suggested that there must be other factors shaping the genetic structure of *N. taihuensis*. Our results were in contrast to the 'Stream Hierarchy Model' [48], which showed that the distribution of genetic variation would follow different basins. Similar results were found in many studies of freshwater fish species [5,9,49,50], including *Opsariichthys bidens *(Teleostei, Cyprinidae) conducted in the Pearl River and the Yangze River in China [8]. However, some researchers [10,11,16] have reported population genetic structure in some freshwater fish species being analogous to our study, and these provided evidence that the contemporary drainage structure did not coincide with the genetic relationships among populations, or that the instream barriers and complex population histories played an important role in the determination of the population genetic structure.

Pair-wise comparisons between *Φ*_*ST *_and AMOVA analysis confirmed the existence of some degree of restricted gene flow, which could be one of the important factors determining the genetic structure among populations of *N. taihuensis*. However, the Mantel test indicated no significant relationship between genetic differentiation and geographic distance among populations. The discrepancy between these results indicated that the pattern of genetic differentiation for *N. taihuensis *was complex and could not be interpreted simply using the IBD model.

In contrast, NCA analysis revealed that multiple unrelated long-distance founding events could be important in determining the patterns of genetic diversity. In NCA, the large *Dn *and small *Dc *suggests long distance dispersal [16]. The NCA inferences for widespread clade 1–6, including the most probable ancestral haplotype H36 with a significantly small *Dc *and large *Dn*, were indicative of range expansion and long-distance colonization (and/or past fragmentation) [51]. The observed population relationships agreed with the population structure predicted by normal dispersal models of recently expanding populations, with a portion of individuals migrating beyond neighbouring demes because of rare long-distance migrants founding pocket populations under this dispersal scenario [16]. Also, theoretical study has proven that even though no effectively restricting gene flow exists under these conditions, the effects on these founder-events can persist for thousands of generations, which results in higher levels of divergence within than between rivers [4]. In addition, it was also suggested that historical basin rearrangements might form the analogous population genetic structure mentioned above [10,16]. Although a strong uplifting of the Tibetan plateau occurred during the Pleistocene in southwest China [31], there is no evidence to indicate that large-scale river rearrangement occurred after the Pliocene in our study area [32]. It may imply that historical basin rearrangements are not critical in determining the current population genetic structure of this species. Accordingly, we suggested that the current complex population genetic structure of this species was mainly constituted by multiple unrelated founding events (long-distance colonization) of dispersal, and also affected by contiguous population expansion and some degree of restricted gene flow.

### Demographic history

Statistical analysis of sequences was originally developed to test selective neutrality of mutations and has recently been used to test for population expansion. Demographic events are also commonly analyzed by using the distribution of pair-wise differences or mismatch distributions of non-recombining DNA sequence such as mtDNA [52,53]. Comparative studies on the test power of various statistics suggested the superiority of *Fs *and *R*_2 _over other statistics [40,41]. The power of these tests also depends on sample size, and the *R*_2 _test is more powerful for a small sample while *Fs *is more powerful for a large sample [41]. In this study, *Fs *tests were significant for the entire region, Yangze River basin and Huai River basin. However, *R*_2 _tests were significant for the entire region and Huai River basin, but not significant for Yangze River basin (Table 5). The different results for population expansion in this study may reflect the differences in sample size requirements of the tests as well as source information.

Mismatch distribution analysis was a common method to infer population demography; however, some of inferences of the mismatch analysis were not consistent with the results of the other indices in this study (Table 5). Besides a relative lower statistical power of demography analysis [41], other factors might also induce such scenario. First, the single stepwise expansion model in mismatch analysis might be inadequate for some populations [54], and the use of an incorrect demographic model can also lead to biased and invalid estimates of demographic history and other evolutionary parameters [55]. As the case for Huai River basin in this study, the skyline plot provided evidence that the exponential growth model could be more realistic than that of stepwise expansion (Figure 4). Second, mismatch analysis needed a pair of genes being chosen at random from the population. In populations having gone through a recent and still large expansion, the internal branches should be very short due to the star-like structure of the tree, and a very few mutations would accumulate on those branches [40,52]. In this case, pairs drawn from the sample would be not independent due to the shared portions of their genealogy [56]. In this study, both unrooted ML tree and the statistical parsimony network displayed "star-like" shape with no deep branching among halotypes (Figure 2, Figure 3). So the inadequate information inferred from mismatch analysis might attribute to the sign in the data set being swamped by non-independence in this study. Moreover, although the unrooted ML tree indicated that there were no distinct haplotype groups with high bootstrap support, the results of our phylogeographic analysis revealed that there were high levels of genetic differentiation among populations, and that the genetic pattern of this species was mainly constituted by multiple unrelated founding events. So cases of founder events not containing all genetic types in some regions might be unavoidable, and could severely affect the shape of the mismatch distribution. Finally, other factors such as high frequency of the ancestral haplotypes, population substructure, or inbreeding could also affect the shape of the mismatch distribution, but to an extent these effects had not been quantified [56].

However, the methods of mismatch distribution analysis and neutrality test did not make full use of the data [57]. The coalescent-based method, by incorporating information from the genealogical tree structure of DNA sequences, used more information in the data and seemed to be more adequate to estimate demographic growth [43,57,58]. In Yangze River and Huai River basin, three different coalescent-based methods, the skyline plot [38,39], the estimate of population growth rate (*g*) using FLUCTUATE 1.4 [43] and the distribution of mutations conducted by GENETREE [44], which provided the similar information of population expansion in these regions, suggested that the superiority of the coalescent-based method. In conclusion, despite of some inconsistency in these results, it was evident that the combined demographic analysis could confirm population expansion in Yangze River and Huai River basin, while a relative constant population size in Pearl River basin.

During the last glacial maximum (LGM, about 18 kyr ago), with sea levels lowered (120–140 m below present sea level) [12], the present distribution range of *N. taihuensis *was almost completely exposed and eradicated, and this species should have been severely impacted by the past glaciations. Our estimate of TMRCA in the Pearl River and the Huai River basin, and the population expansion time for *N. taihuensis *in the Yangze River basin were much older than that of LGM. It was difficult to link the population demographic history to any particular Pleistocene paleo-climatic event. However, the gene tree (Figure. 6) provided evidence that the Pleistocene ice ages had a great effect on the demographic history of this species. That the vast majority of mutations occurred near the tips of the gene tree suggests that population expansion events mainly occurred after LGM. Therefore, we proposed that in the Yangze River basin and the Huai River basin, the *N. taihuensis *should have been the most severely impacted by the past glaciations, surviving in some refugium, and then post-glacial colonizations and population expansions occurred with the climate warming and sea levels uplifting after the late Quaternary (especially the LGM) [59]. Further, three near-coastal populations (the Taihu lake population and the Chaohu population in the Yangze River basin, the Hongzehu Lake population in the Huai River basin) contained higher numbers of haplotypes, haplotype diversity (*h*) and nucleotide diversity (*π*) (Table 1), suggesting that a glacial refugium could exist near the estuaries. However, *N. taihuensis *has been exposed to pollution and habitat destruction for decades; its population size has decreased drastically in out-flowing rivers and estuaries of these basins under study [24,25]. No sample from these locations could be collected to detect the probable glacial refugium. Further studies should be conducted in future. In addition, it is worthy to note that many attached lakes formed in these areas with sea levels uplifting after the LGM. For example, Chaohu Lake and Taihu Lake in the Yangze River basin shaped about 12 and 2.6 kyr ago, respectively, and Hongzehu lake and Weishanhu lake in the Huai River basin was shaped about 1.4 and 0.6 kyr ago, respectively [60]. This indicated that the formation of appropriate habitations facilitated historical population expansions. This inference was compatible with the "r-strategy" characteristic of *N. taihuensis *with high relative fertility and a shorter generation time [29], which also suggests its population size could be severely affected by climate and habitation. Thus, it is not surprising that *N. taihuensis *has maintained a relatively constant population size in the Pearl River basin, because this area is a subtropical zone, with average 2 – 3°C higher than in the Yangze River basin or the Huai River basin during the Pleistocene [61]. There was no large-scale creation of appropriate habitations (attached lakes) after the LGM in this area.

### Implications for Conservation

The Salangid, *Neosalanx taihuensis*, is one of the most important commercial species in this family. The dry or frozen salangids are good materials in Chinese food, and they are also exported to Japan and other southern-east Asia countries. But, it has to be noticed that the population size of *N. taihuensis *and its fishing yield have decreased rapidly in recent years [24,25]. For example, the yield of Salangids per year in Poyanghu lake decreased from 6 × 10^5 ^kg in 1960s to 10^4 ^kg in 1987 [62], and the fishery production of *N. taihuensis *was completely collapsed in Poyanghu lake thereafter [63]. The yield of Salangids per year in the Yellow River has also decreased since 1987, and the lowest yield per year less than 10^3 ^kg occurred in 1990 [29]. According to our investigation being from 2004 to 2005 and the information from the local fishery management offices, both adults and juveniles are now rarely found in the Yellow River. Some authors thought that over-fishing was a serious problem [64], but You *et al*. suggested that over-fishing was not the most important threat to this species [65]. On the other hand, the water pollution should be also the one of the most serious threat to *N. taihuensis *[25,63,66]. The environment of *N. taihuensis *has been polluted because of the waste water from urban, industry and agriculture [25]. Finally, the threat to *N. taihuensis *is also from the habitat fragmentation. Since mid 1950s, the aquatic networks have been fragmented dramatically with the construction of dykes and floodgates across the river-lake connections [60,66]. Most of the lakes in the mid-lower reaches lost their connection with the main river watercourse by the mid 1980s [60]. Consequently, most of the small lakes nearly disappeared and the mid-large lakes shrank considerably resulting in unsuitable habitat for this species [66,67]. Up to the present, no specific protect action has been taken for *N. taihuensis*, so it is urgent to make an effective plan to protect this economically important species.

The study on population structure and its phylogeoraphic pattern could provide particularly information for us to more broadly consider conservation options and more accurately assign appropriate fishery management [68]. According to the model proposed by Moritz [69], Evolutionary Significant Units (ESUs) are designated on the basis of reciprocal monophyly at mitochondrial markers. In the present study, phylogenetic inference based on ML and NCA methods revealed that it was no geographical structure among haplotypes in *N. taihuensis*, suggesting that there was not a genetically distinct and independent population that could be considered as an ESU. However, AMOVA analysis indicated that significant genetic variance resulted from genetic differences among populations and within basins. In addition, based on low vagility, geographic isolation among most populations and rapidly population decline over the recent decades for this species [24,25,63], we proposed that all populations should be managed and conserved separately. Moreover, special attention must be paid on habitat protection and pollution control because this species is susceptive to appropriate habitat change.

## Conclusion

Our results showed that this species presented a very random genetic pattern and was not compatible with the present hydrological structure. This genetic pattern might be formed mainly by multiple unrelated long distance founding events, combining with population expansion and the instream barriers restricting gene flow. Demographic analysis revealed that the populations in the Pearl River basin (TMRCA approximately 73.53 kyr) has kept a relatively constant population size of *N. taihuensis*, but populations in the Yangze River basin and the Huai River basin expanded about 221.9 and 190.0 kyr ago, respectively, with the majority of mutations occurring after the LGM. We concluded that the more favourable climate and the formation of appropriate large-scale habitations facilitated this expansion. We suggested that it was necessary to develop a suitable management plan for *N. taihuensis *based on our results.

## Methods

### Sample Collection

We collected a total of 354 individuals of *N. taihuensis *from 13 populations in the Pearl River, Yangtze River, and Huai River basins from March in 2004 to September in 2005, 44 came from two populations of the Pearl River basin in 2005 (P1–P2), 196 from seven populations of the Yangtze River basin in 2004 (Y1–Y7), and 114 from four populations of the Huai River basin in 2005 (H1–H4) (Figure. 1 and Table 1). To eliminate the possibility that these samples came from the related individuals (schools of fishes), we selected many patches from one location and resampled 2–4 times in same population. All samples (whole fish) were stored in 95% ethanol.

### DNA extraction, PCR amplification and Sequencing

Total DNA was extracted from the muscle tissue using standard phenol/chloroform procedures [70] or following the method of Asahida *et al*. [71]. PCR was performed according to the method of Zhang *et al*. [30]. 1141 base pairs (bp) of *cytb *DNA were amplified using the primers L14321 (5'-CAGTGACTTCAAAAACCACCG-3') and L15634 (5'-CTTAGCTTTGGGAGTTAAGGGT-3') in a 50 μl reaction volume mixture containing 25 μl *Premix Taq *(1.25 U EX Taq polymerase, 0.4 mM of each dNTP Mixture, 4 mM Mg2+; TaKaRa, Tokyo, Japan), 1.0 μM of each primer, approximately 50–100 ng total genomic DNA template and 18–22 μl of sterile distilled water. A PE 9700 Thermal Cycler (Applied Biosystems) was used for the following cycles: pre-denaturation at 94°C for 5 min; 30 cycles of denaturation at 94°C for 30 s, annealing at 54°C for 45 s, and extension at 72°C for 1 min 10 s, plus a final extension at 72°C for 10 min. Purified PCR products were directly sequenced using primers L14321 and L15634 from both ends using the Big Dye Terminator Sequencing Kit (Perkin-Elmer, Norwalk, CT) in a semi-automated DNA analyzer (3700; Applied Biosystems). To avoid the errors in amplification and sequencing, all singletons among polymorphism sites were verified by an additional amplification and sequencing. Sequences were aligned using the Clustal X program [72] and rechecked against the inferred reading frame for the corresponding protein. Haplotypes were identified using Clustal X, and the *cytb *haplotype sequences of *N. taihuensis *were deposited in GenBank (EU376454–EU376489).

### Data Analysis

#### Genetic Diversity and Population Structure

In order to test whether there is recombination within *cyt b *gene, linkage disequilibrium (LD) test was conducted using DnaSP 4.10 [73] and the average degree of LD, or non-random association between nucleotide variants at different polymorphic sites was tested using ZZ statistics [74], which could be used for detecting intragenic recombination with the null hypothesis no recombination. Also, neutrality test for *cyt b *was conducted in MEGA4.0 [75] to check selection by Z-test using the null hypothesis H0: d_N _= d_S _[76,77], where d_S _and d_N _is the average number of synonymous substitutions per synonymous site and the average number of nonsynonymous substitutions per nonsynonymous site, respectively.

Genetic diversity was measured for all samples and for each basin grouping using haplotype diversity (*h*) and nucleotide diversity (*π*) [78]. Values for the numbers of haplotypes (*H*), polymorphic sites (*S*) and the mean numbers of pair-wise differences among sequences (*K*) were also estimated. These diversity indices were computed using the software DnaSP 4.10 [73]. As unequal numbers of individuals varying from 13 to 34 per population in this study, Pearson's correlation tests were conducted to test values independence of the number of haplotypes (*H*), haplotype diversity (*h*) and nucleotide diversity (*π*) against the number of individuals (*N*). Population structure and genetic variation of *N. taihuensis *were characterized and compared with Arlequin version 3.11 [79]. Analysis of molecular variance (AMOVA) was used to asses the population configuration and the geographical pattern of population subdivision. In this study, populations were grouped according to different geographical hierarchies using *Φ*-statistics [80]. Three hierarchical levels of subdivision were obtained: *Φ*_*CT*_, the degree of differentiation among all basins, *Φ*_*SC*_, the degree of differentiation among populations within basins, and *Φ*_*ST*_, the degree of differentiation among all populations. We tested whether the derived indices were significantly different from zero using a nonparametric permutation method (10,000 Permutations). Moreover, we estimated pair-wise genetics differentiation between populations with *Φ*_*ST *_[80,81] that includes information on haplotypes frequency and information on haplotype sequences. Null hypothesis of genetic homogeneity was assessed by 10,000 replications and sequential Bonferroni corrections [82] for multiple comparisons were applied to all pairs comparisons. To verify the hypothesis of isolation-by-distance (IBD), correlation between pair-wise linearized Φst/(1-Φst) and river distances (Km) between populations were analyzed using the Mantel test [83] with 10,000 permutations. Mantel test were conducted for all 13 populations among basins, for 7 populations within Yangze River basin and for 4 populations within Huai River basin, respectively. The river distances between populations were determined based on river courses by AcrView 3.2, which was also used in NCA analysis.

#### Phylogenetic and Phylogeographic Analysis

For phylogenetic analysis, we constructed unrooted maximum-likelihood (ML) tree using the program PAUP* 4.0 [84]. The TrN+I (I = 0.8148) model was selected as the best-fit model for analysis using MODELTEST 3.06 [85].

Intraspecific data typically consists of many similar sequences, some of which may be ancestral and its phylogenetic relationship are often more clearly and accurately represented by a network [86]. In the present study, we constructed a haplotype network based on statistical parsimony [87] using the program TCS version 1.18 [88]. A distance matrix for all pair-wise haplotype comparisons was constructed and the maximum number of mutational differences justified by the parsimony limit of 0.95 was estimated. The network first constructed haplotypes that differed by a single change and added increasingly more distant haplotypes until either all were included or the maximum number of mutational steps was reached [88]. To check the evolutionary mechanisms responsible for the spatial distribution of genetic variation, we conducted the Nested Clade Analysis (NCA). The resulting cladogram calculated by the TCS program was converted into a nested design using the nesting rules [89]. Reticulations, or equally parsimonious connections within the network, were resolved with two steps. First, alternative connections between haplotypes were broken following a series of rules based on coalescence theory [90]. Second, synapomorphies in the form of nonsynonymous substitutions in the *cytb *gene, a conservative class of substitutions, was used to resolve all cases in which assignment to a nested series was ambiguous [89]. The current and historic patterns of phylogenetic and geographic associations were statistically tested using NCA implemented in GeoDis 2.0 [91], under the null hypothesis of no geographic association among haplotypes using 10,000 permutations. The most suitable model explaining the historical geographical distribution was identified using the inference key [51]. It is appropriate to address the caveats of NCA analysis, the power of this method has been criticized for years [92-94], but Templeton defended NCA on both theoretical and empirical grounds, and insisted this method an extensively validated method for strong phylogeographic inference [51,95]. However, as no other better all-encompassing method can offer the ability to explore patterns relating to complex historical scenarios at present, the NCA method can still provide useful complementary information for phylogeographical analysis [96].

#### Demographic Analysis

To detect mechanisms responsible for observed mtDNA patterns on various spatial and time scales, we developed different data analysis methods. Due to the limits and pitfalls of each method, the combined results might provide the best approximation of the population structure and history.

First, changes over time in nonparametric estimates of the effective population size of *N. taihuensis *were evaluated with the skyline plots [38,39] using R 2.6.1 [97]. This method can provide a rough profile of population demographic fluctuation, and can be used as a model selection tool. The classic skyline plot typically produces 'noisy' plots that display the stochastic variability inherent in the coalescent process. While using the Akaike Information Criterion [98], the generalized skyline plot could reduce this noise, and thus produce smoother estimated population size plots [38]. As the power of skyline plots depends on the numbers of variable nucleotide sites [38,55], we conducted skyline analysis for the entire region, the Yangtze River basin and the Huai River basin with exception of the Pearl River basin. Second, for the entire region and for each basin, we examined the observed distribution of pair-wise differences between sequences (mismatch distribution) [53]. Theoretical studies show that mismatch distributions are usually ragged or multimodal for populations at stationary demographic equilibrium, but are typically smoother or unimodal for populations that have recently undergone a demographic expansion [53]. Occasionally, a pronounced rate of heterogeneity is reported even among closely related lineages [59], so the pair-wise relative rate test (pRRT) implemented in HyPhy [99] was used to estimate and verify molecular clock constancy at the intraspecific level based on 1141 bp of 36 haplotypes and *P. chinensis *(DQ191115) [30] before performing the mismatch distribution analysis. One haplotype (H31) was identified to evolve at a significantly different pace from the others. After removing this haplotype (H31), no other violation of the molecular clock constancy was found, so the other 35 haplotypes were used for further analysis. Mismatch analysis was conducted using Arlequin 3.11 [79] under a model of population expansion. The overall validity of the estimated demographic model was evaluated by the tests of raggedness index (*Hri*) [100] and the sum of squared differences (*SSD*) [101]. Significance of *Hri *and *SSD *were assessed by parametric bootstraps (10,000 replicates), and the significant value was taken as evidence for departure from the estimated demographic model of sudden population expansion. Third, Fu and Li's D* [42], Fu's *Fs *[40] and Ramos-Onsins and Rozas's *R*_2 _[41] tests for mutation/drift equilibrium were performed in DnaSP 4.10 [73] and Arlequin 3.11 [79] with 10,000 simulations. Although these methods are commonly used to test the selective neutrality of genetic markers, these estimators are also sensitive to demographic processes such as recent population expansion or bottleneck. Because we were interested in discriminating between demographic expansion and contraction, we chose two class test statistics, each with particular sensitivity to one demographic scenario. Fu and Li's D* is designed to detect an excess of old mutations, characteristic of a population that has experienced a historical reduction in effective population size [42,102]. In contrast, Fu's *F*_*S *_and *R*_2 _are sensitive to an excess of recent mutations [40,41]. Finally, we used the coalescent-based method implemented in FLUCTUATE 1.4 [43] to estimate exponential growth rate (*g*) for this species. All runs employed the following strategy: 10 short chains of 4,000 steps and five long chains of 400,000 steps, sampling every 20th step; random starting trees; empirical nucleotide frequencies; initial *g *value of 0.0, starting *θ*-value from Watterson's estimate [103]; and a 2.424 transition/transversion (ti/tv) rate determined from MEGA4 [75] under the Tamura-Nei model [104] using only ingroup sequences. Runs were repeated five times to ensure consistency of estimates. As the estimates of *g *are biased upwards [43], a conservative approach in testing for significance was adopted in our study, with values larger than three standard deviations (*SD*) of *g *regarded as significant.

For stepwise expansion population, we used *τ *to calculate the expansion time. The mismatch distribution analysis provided a rough estimate of *τ*, with the starting time of the expansion in units of 1/(2 ut) generations, where u is the mutation rate per locus per generation. The relationship with the absolute time in years (t), is t = *τ*/2 uT. The value of T is generation time (1 year for *N. taihuensis*), and the value of u is derived from u = μk, where μ is the mutation rate per nucleotide per generation, and k is the number of nucleotides in the sequence. For other populations, however, we used GENETREE [44] to estimate the time to the most recent common ancestor (TMRCA). GENETREE is based on a coalescent method to simulate gene trees conditional on their topology, which can be used to determine the distribution of TMRCA. In present study, the most probable ancestral haplotype was analyzed by TCS program, and the TMRCA for a constant population was calculated in GENETREE by first finding the maximum-likelihood estimate [105] of *θ *following the method of Joy *et al*. [106]. Each run was repeated three times with a different starting seed number and 1,000,000 coalescent simulations. To get a more detailed picture of the mutational pattern from population expansion, GENETREE was also used to construct a gene tree to determine the distribution of each mutation for *N. taihuensis*.

Since there is no fossil record for *N. taihuensis *to analyze their cartilage character, the genetic time clock is difficult to calibrate. However, it has been convincingly shown that most groups of fish have slower evolutionary rates compared with those of other species, for example primates [35]. A calibration rate of 0.8–1% per Myr per generation has been accepted for mitochondrial protein-coding genes in Salmoniformes or Osmeriformes in many studies [70,107,108]. For convenience, we used the mutation rate μ = 1% in this study.

## Abbreviations

AMOVA: Analysis of molecular variance; ML: maximum likelihood; NCA: nested clade analysis; IBD: isolation by distance; LGM: last glacial maximum; TMRCA: the most recent common ancestor.

## Authors' contributions

LZ collected samples, isolated genomic DNA, and conducted the amplification and sequencing of the complete Cyt *b *gene. He collected, analyzed and summarized the data, and drafted the manuscript; JZ collected some samples, contributed some sequencing and contributed some to its design; ZL participated in analysis and interpretation of data, and helped draft the manuscript; SMF revised the manuscript and conducted part of analysis; FW and MX revised the draft manuscript and gave their some comments; ML conceived the study and participated in its design and data interpretation, and preparing the manuscript. All authors read and approved the final manuscript.

## Supplementary Material

Additional file 1 Nested clade analysis. Statistical analysis of the current and historic patterns of phylogenetic and geographic associations. Click here for file
