# Supplementary material for: Complex population genetic and demographic history of the Salangid, Neosalanx taihuensis, based on cytochrome b sequences
Source: BMC Evol Biol. 2008 Jul 14;8:201. doi: 10.1186/1471-2148-8-201 (PMC2483725; doi:10.1186/1471-2148-8-201)
Supplement: Additional file 1 — Nested clade analysis. Statistical analysis of the current and historic patterns of phylogenetic and geographic associations. [file 1471-2148-8-201-S1.doc]

**Additional file 1：** Nested clade analysis

| Nested clade | P | Interior clades | *D*_C_ | *D*_N_ | Inference |
| --- | --- | --- | --- | --- | --- |
| Clade 1-2 | 0.000 | clade H13 ( Tip ) | 277.96 | 305.83 | 1-19-20-2-11-17-4 RGF |
|  |  | clade H14 ( Tip ) | 296.52 | 329.58 |  |
|  |  | clade H33 ( Interior ) | .478.33 | 399.54L |  |
|  |  | I-T | 193.85 | 85.37 |  |
| Clade 1-4 | 0.000 | clade H02 ( Tip) | 0.00 | 1147.64L | 1-2-3-4 RGF |
|  |  | clade H07 (Tip) | 368.30S | 530.60 |  |
|  |  | clade H25 (Tip) | 255.01S | 464.67 |  |
|  |  | clade H27 ( Interior ) | 430.72 | 531.47 |  |
|  |  | I-T | 135.46 | -0.372 |  |
| Clade 1-6 | 0.000 | clade H01 (Tip) | 0.00 | 0.00 | 1-2-3-5-6-13 LDC |
|  |  | clade H08 (Interior) | 182.22S | 528.09S |  |
|  |  | clade H09 (Tip) | 0.00 | 0.00 |  |
|  |  | clade H10 (Tip) | 185.52S | 544.58S |  |
|  |  | clade H20 ( Tip) | 0.00 | 0.00 |  |
|  |  | clade H21 (Tip) | 836.72L | 814.36L |  |
|  |  | clade H03 (Tip) | 330.72 | 614.76 |  |
|  |  | clade H05 ( Tip) | 94.87S | 488.87S |  |
|  |  | clade H06 (Tip) | 0.00 S | 674.08 |  |
|  |  | clade H16 (Tip) | 0.00 | 0.00 |  |
|  |  | clade H17 ( Tip) | 0.00 S | 516.18S |  |
|  |  | clade H31 (Tip) | 985.63 | 679.31 |  |
|  |  | clade H34 (Tip) | 521.96 | 795.27L |  |
|  |  | clade H30 ( Tip) | 111.31S | 509.08S |  |
|  |  | clade H22 (Tip) | 0.00 S | 523.40S |  |
|  |  | clade H23 (Tip) | 269.07S | 642.55 |  |
|  |  | clade H24 (Tip) | 194.90 | 489.55S |  |
|  |  | clade H26 (Tip) | 0.00 S | 536.22S |  |
|  |  | clade H28 (Tip) | 0.00 | 644.65 |  |
|  |  | clade H36 (Interior) | 0.00 S | 889.08L |  |
|  |  | clade H29 (Tip) | 272.08S | 574.94S |  |
|  |  | I-T | -278.99S | 268.09L |  |
| Clade 1-10 | 0.000 | clade H19 (Tip) | 910.09 | 762.25L | 1-19-20-2-11 RE |
|  |  | cladeH35 (Interior) | 134.05S | 457.75 |  |
|  |  | I-T | -776.04 | -304.50 |  |
| Clade 2-1 | 0.000 | clade 1-1 (Tip) | 281.36S | 396.68 | 1-2-3-4RGF |
|  |  | clade 1-2 (Interior) | 407.98 | 475.39L |  |
|  |  | clade 1-3( Tip ) | 484.10 | 481.93 |  |
|  |  | I-T | 111.61 | 72.40L |  |
| Clade 2-2 | 0.000 | clade 1-4 ( Tip ) | 429.53 | 428.59 | 1-2-3-11-17-4 RGF/IBD |
|  |  | clade 1-5 (Interior) | 498.26 | 443.23L |  |
|  |  | I-T | 68.73 | 14.65L |  |
| Clade 2-3 | 0.000 | clade 1-6 (Interior) | 415.32S | 535.01L | 1-2-3-5-6-14 LDC/PF |
|  |  | clade 1-7 (Tip) | 107.70S | 381.06 S |  |
|  |  | clade 1-8 (Tip) | 478.93 | 609.77L |  |
|  |  | clade 1-9 (Tip) | 243.52S | 396.94S |  |
|  |  | clade 1-10 (Tip) | 239.38S | 558.13L |  |
|  |  | I-T | 149.64L | 41.51L |  |
| Total  cladogram | 0.000 | Clade 2-1 (Tip) | 421.95S | 482.18S | 1-2-11RE-12 CRE |
|  |  | Clade 2-2 (Interior) | 367.44S | 442.29S |  |
|  |  | Clade 2-3 (Tip) | 660.68L | 604.98L |  |
|  |  | I-T | -178.87 S | -103.86  S |  |

Note: Clade (*D*_C_) and nested clade (*D*_N_) distances are given for all nested clades containing geographic and genetic variation. An “S” indicates that the distance is significantly small (p<0.05), and an L indicates that it is significantly large (p < 0.05). Interior versus tip distances, *D*_C_ and *D*_N_ are given for nested clades where the interior/tip status is known and for which both tips and interiors exist. Within the same nesting group, the clade name is shaded for interior clades. Inferences are given following the Templeton *et al.* (2004) inference key. PF- past fragmentation; RGF - restricted gene flow; RE - range expansion; RGD - restricted gene dispersal; LDC- long-distance colonization; PF-past fragmentation; CRE - continuous range expansion.
